# Tissue-wide cell-specific proteogenomic modeling reveals novel candidate risk genes in autism spectrum disorders

**DOI:** 10.1038/s41540-022-00243-8

**Published:** 2022-09-06

**Authors:** Abolfazl Doostparast Torshizi, Kai Wang

**Affiliations:** 1 Raymond G. Perelman Center for Cellular and Molecular Therapeutics, Children’s Hospital of Philadelphia, Philadelphia, PA 19104 USA; 2 Department of Pathology and Laboratory Medicine, Perelman School of Medicine, University of Pennsylvania, Philadelphia, PA 19104 USA

**Keywords:** Systems biology, Regulatory networks

## Abstract

Autism spectrum disorders (ASD) are a set of complex neurodevelopmental diseases characterized with repetitive behavioral patterns and communication disabilities. Using a systems biology method called MAPSD (Markov Affinity-based Proteogenomic Signal Diffusion) for joint modeling of proteome dynamics and a wide array of omics datasets, we identified a list of candidate ASD risk genes. Leveraging the collected biological signals as well as a large-scale protein-protein interaction network adjusted based on single cell resolution proteome properties in four brain regions, we observed an agreement between the known and the newly identified candidate genes that are spatially enriched in neuronal cells within cerebral cortex at the protein level. Moreover, we created a detailed subcellular localization enrichment map of the known and the identified genes across 32 micro-domains and showed that neuronal cells and neuropils share the largest fraction of signal enrichment in cerebral cortex. Notably, we showed that the identified genes are among the transcriptional biomarkers of inhibitory and excitatory neurons in human frontal cortex. Intersecting the identified genes with a single cell RNA-seq data on ASD brains further evidenced that 20 candidate genes, including *GRIK1*, *EMX2*, *STXBP6*, and *KCNJ3* are disrupted in distinct cell-types. Moreover, we showed that ASD risk genes are predominantly distributed in certain human interactome modules, and that the identified genes may act as the regulator for some of the known ASD loci. In summary, our study demonstrated how tissue-wide cell-specific proteogenomic modeling can reveal candidate genes for brain disorders that can be supported by convergent lines of evidence.

## Introduction

Autism spectrum disorders (ASD) are a group of neurodevelopmental disorders predominantly characterized with behavioral and communication impairments. According to the American Psychiatric Association, autistic individuals share a spectrum of symptoms including difficulties with interactions with other people, repetitive behaviors, and inabilities to function properly in a daily life^1^. According to the US Centers for Disease Control and Prevention^2,3^, 1 out of 54 children are diagnosed with ASD across the US. ASD are known for extreme heterogeneity in their genetic components^4–6^ as well as 4:1 ratio of prevalence among men compared to women^7,8^. Such pervasive disorders can be attributed to distinct phenotypic subtypes. Accumulating evidences suggest that myriads of genetic signatures of ASD might converge to a tractable set of pathways or gene networks for effective therapeutic practices^9,10^. At genome level, common variants identified by genome-wide association studies^11,12^ as well as other genetic perturbations such as copy number variants, de novo mutations, and function-disrupting point mutations account the ASD liability^13,14^. Thus far, large-scale genome-wide studies such as iPSYCH project, with thousands of participants^13^, have brought about outstanding insights into the genetic architecture of ASD. However, there exists significant challenges to address such as spatial distribution and enrichment patterns of genetic hits across brain, cellular specificity of distinct brain regions, and complementary role of less studied omics data such as proteome granted that significant fraction of studies have focused on nucleic acids, i.e., genomics, epigenomics and transcriptomics rather than amino acids^15^.

Genetic association studies, such as GWAS, CNV, whole genome, and exome sequencing^6,14,16,17^, have remarkable impact on pinpointing genes and genetic variants associated with ASD. Moreover, transcriptomic^18–20^ and proteomic studies^21,22^ have significantly contributed to our understanding of the disease machinery. However, there is no clear agreement between gene expression levels and the abundance of their corresponding encoded proteins^15^ given that, at best, there is a 40–60% correlation between the mRNA and protein levels in certain organisms^23,24^. Such disagreements may arise from the fact that functionality of proteins are not solely determined by their abundances but by other biochemical or biophysical properties such as post-translational modifications and subcellular localization^25^. In spite of fascinating progresses, critical questions remain unanswered: are there any converging mediums to illustrate the confluence of small effect sizes of all of the loci, considering proteins as functional components of cellular machinery, how can we model biochemical and biophysical properties of proteins in parallel with genomic information to mimic the real circuitry of the disease and unmask novel loci which may participate in ASD development?, how we can create such a system level picture at wide range of cell-types across distinct brain regions?. Addressing these questions requires integrated mechanisms. Prikshak et al.^10^ had mapped ASD risk genes onto co-expression networks aimed at recovering developmental trajectories which represent fetal and adult cortical luminae. Notably, they had reported how FMRP (encoded by *FMR1*) regulation as well as co-expression of common transcription factors connect synaptic development with early transcriptional regulation^10^. Recently, Ramaswamy et al.^26^ reported an integrative study leveraging mRNA and miRNA expression, DNA methylation, and histone acetylation from ASD and control brains to uncover convergent molecular subtypes of ASD. Their research has led to a substantial expansion in the repertoire of differentially expressed (DE) genes as well as identification of highly enriched hyper-acetylated noncoding genomic regions. Additional integrated studies combining protein-protein interactions^27^ and gene expression suggest molecular convergence in subsets of ASD risk loci^28–30^. Yet, it remains an open question on how intra-cellular molecular interactions and protein trafficking paradigms within the cell in certain central nervous system (CNS) regions can be employed to identify highly-specific convergent gene modules to contribute to ASD etiology.

To address these questions, we have employed our develop method called MAPSD^31^, Markov Affinity-based Proteogenomic Signal Diffusion, for joint modeling of biochemical and biophysical properties of proteins with genomic and transcriptomic signatures of ASD aimed at identification of novel ASD candidate risk genes. Upon collecting multiple sources of omics data from the literature, MAPSD is able to generate tissue and cell-specific interactomes followed by diffusing the known biological signals throughout the protein-protein interaction networks (PPI) networks in order to predict unannotated susceptible genes. MAPSD is designed to be used in complex diseases where a wide array of biological data is available. A good example of such diseases is schizophrenia (SCZ) in which high polygenicity of the disease has been extensively studied in the literature^32^. Our previous study on SCZ had revealed a shortlist of novel disease associated candidate risk genes with a similar enrichment patterns in specific cellular micro-domains in neuronal cells in human cerebral cortex^31^. Given the availability of valuable resources including distinct molecular data on ASD, this is a great opportunity to account for the complexities of ASD through a novel systems-level approach. Our findings indicate the importance of parallel consideration of omics data-types in improving the understanding of the genetic architecture of ASD.

## Results

### Application of MAPSD to identify candidate genes for ASD

**Fig. 1:**
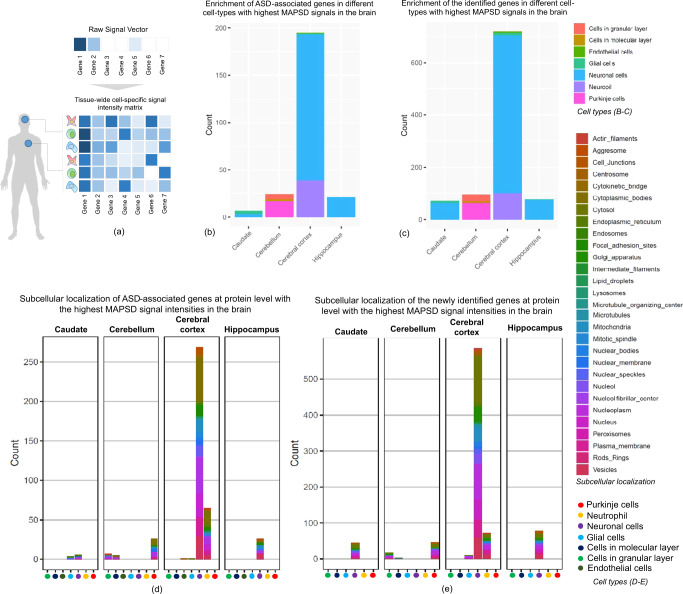
A schematic of MAPSD and Enrichment patterns of MAPSD brain-specific gene-encoded proteins at cell resolution and subcellular domains using the Human Protein Atlas data. **a** A schematic of MAPSD which starts with raw signal vector of a gene list and diffuses them onto 131 combinations of tissues and cell-types given the structural topology of the PPI network and biochemical and biophysical properties of proteins. Darker cells represent high intensity genes regarding a specific cell, vice versa; **b** Enrichment of MAPSD original ASD risk genes at single cell resolution in four brain regions; **c** Enrichment of MAPSD newly identified ASD risk genes at single cell resolution in four brain regions; **d** Enrichment of MAPSD original ASD risk genes is various subcellular domains in five cell-types across four different brain regions; **e** Enrichment of MAPSD newly identified ASD risk genes is various subcellular domains in five cell-types across four different brain regions.

We used MAPSD^31^, a multi-omics data integration method, to identify additional candidate genes for ASD from existing knowledge. MAPSD first identifies omics information (genome, transcriptome, proteome) on known candidate genes for a specific disease from various sources; it then receives PPI networks, subcellular localization of proteins within cellular micro-domains, and protein abundances across >130 different combinations of tissues and cell-types as well as a repertoire of other omics data-types followed by diffusing the accumulating signal intensities of available genetic signatures through tissue/cell-type adjusted PPI networks, to uncover disease-relevant genetic drivers of the disease with small effect sizes which cannot be captured using available single-omics pipelines (Fig. 1a). In fact, MAPSD leverages dedicated properties of proteins to initially adjust the PPIs given the tissue or cell-type being studied.

To create the PPI, we assembled the PPI networks from three sources including: PICKLE 2.3^33,34^, The Human Reference Interactome^27^, and human Interactome Database^35^. Upon removing duplicate interactions, 232,801 interactions were used in the study. Four layers of omics data on ASD were collected including genome, epigenome, transcriptome, proteome, and connectome (see Methods and Materials, Fig. 1a). We used the SFARI gene list to obtain the genome data containing 1089 genes which have been curated from the literature by human experts, where each gene has varying degree of evidence to be associated with ASD. The genome data consists of genetic mutations being categorized into four groups based on SFARI gene set including: common variants, rare variants, syndromic variants i.e., genes implicated in syndromic forms of ASD where only a subpopulation of patients develop autistic symptoms, and functional mutations, i.e., ASD candidate genes not covered by the other categories whose occurrence manifests autistic symptoms. As a part of PsychENCODE consortium, Gandal et al.^36^ have drawn a map of transcriptome-wide isoform-level dysregulated genes in ASD. We included 1611 DE genes and 767 differentially spliced genes. MAPSD requires an initial signal vector which is a collection of ASD-associated risk loci from any of the omics data layers used. For example, if a gene is DE and differentially methylated, then its initial signal value will be 2. The initial signal vector used in MAPSD included 3205 genes with signal intensities ranging from 1 to 4. 78% of the initial ASD loci had an initial signal of 1 while only 1.2% had an initial signal level of 4 (Supplementary Table 1). Some of the loci with significant signal intensities include: *SHANK2*, *SHANK3*, *RORA*, and *GRIN2A*. We note that all the datasets curated in this study are adjusted for sex to avoid any biases in the results.

The biochemical and biophysical properties of proteins being used in this study were obtained from the Human Protein Atlas^37,38^. This data is twofold: first, the protein abundances across 131 tissues/cell-types from normal humans; second, tissue-wide subcellular localization of proteins within intra-cellular micro-domains (Supplementary Fig. 1a). Projecting subcellular localization information onto the PPI networks creates a more realistic image of intra-cellular trafficking of proteins given that the likelihood of interactions between two molecules residing in the same micro-domain is higher than the ones localized far from each other. On the other hand, the abundance of proteins at cell resolution in tens of different tissues enables us to adjust the topology of PPI networks and create a resilient network structure dedicated to specific tissues and cell-types. Given the information in the Human Protein Atlas^37,38^, 32 subcellular micro-domains were used in this study (Supplementary Fig. 1b). Some of the micro-domains where a large number of proteins are expressed include cytosol, nucleoplasm, nucleus, and plasma membrane. MAPSD receives all of these information and diffuses the disease signals from known ASD loci to the entire human interactome each adjusted for the given tissue being studied. In our study, four specific brain regions were targeted including: cerebral cortex, cerebellum (CB), caudate, and hippocampus. We executed MAPSD and checked the initial ASD loci showing the highest signal intensity in various brain regions. 247 genes were found to show high signal intensities uniquely in the brain after signal diffusion (Supplementary Table 2).

### MAPSD-identified risk genes-encoded proteins are enriched in specific subcellular domains in neuronal cells

Executing MAPSD resulted in a set of 1209 genes which showed the highest signal intensity in the brain. Of which, 247 genes were ASD-associated genes initially fed to MAPSD and 962 were newly identified. We separated the two gene sets and investigated their spatial enrichment in the brain within the Human Protein Atlas. We observed that 154 ASD genes (62%) were highly enriched in neuronal cells within cerebral cortex while cerebral cortex shared the largest fraction of ASD risk genes (~80%, Fig. 1b). We sought to evaluate the set of the newly identified genes in the brain. Out of 962 new susceptibility disease risk genes, 719 genes (~75%) were enriched in all of the cell-types in cerebral cortex where 605 genes were specifically enriched in neuronal cells within this region (~63% of the entire genes, Fig. 1c). Notably, these observations reveal an agreement between the enrichment patterns of the both gene sets and ensures reliable cell-specificity of MAPSD.

We were interested in finding where ASD risk genes localize in subcellular micro-domains. We used subcellular localization data from the Human Protein Atlas^37,38^. First, checking the ASD loci with the highest MAPSD signal in the brain, we observed significant enrichment of the disease susceptibility risk loci in neuronal cells in cerebral cortex (Fig. 1d). 82% of these loci were enriched in different cell-types across cerebral cortex. Among which, five subcellular micro-domains harbored ~54% of the entire loci essentially enriched in neuronal cells including: cytosol (16%), nucleoplasm (13%), and nucleus (9%), plasma membrane (7%), and vesicles (8.6%). This is a strong indication that not all of the domains within a cell are disrupted by the disease. Similarly, in the newly identified gene set, ~78% of the identified loci were enriched across various cell-types in cerebral cortex. 46% of these loci were enriched in specific cellular micro-domains including cytosol (16%), nucleoplasm (11%), nucleus (6%), plasma membrane (6%), and vesicles (7%) (Fig. 1e). We checked if there were any differences between the obtained enrichment proportions in the both gene sets. No significant difference between the proportions of enrichment percentage in cytosol, nucleoplasm, nucleus, plasma membrane, and vesicles were observed (two-sample *z*-test at significance level of 0.05). This observation supports how the identified genes and their protein products are similarly localized within subsets of cell-types in the brain compared to ASD risk genes. Some of these observations have been made in autistic individuals. For example, a study by Beheshti et al.^39^ shows how *ASTN2*, a gene associated with ASD through disruptive copy number variation, binds to surface of synaptic proteins affecting their trafficking leading to the modulation of synaptic strength. In addition, certain subcellular micro-domains to show a strong association with the disease in our study have been corroborated to be implicated in ASD. For instance, plasma membrane^40^, nucleus^41^, and cytosol^42^ have been recognized to implicate in ASD. We were interested to conduct a functional enrichment analysis to check what biological pathways are enriched for the set of the identified candidate risk gene. Using WebGestalt^43^, we performed a pathway enrichment analysis. We observed a number of pathways significantly enriched including: Neuroactive ligand-receptor interaction (FDR = 3.4e−14, Ratio: 3.5), SNARE interactions in vesicular transport (FDR = 0.01, Ratio: 1.6), and Glutamatergic synapse (FDR = 0.027, Ratio: 1.7). In fact, these pathways have been previously found to be involved in ASD^44–46^ providing further evidence on potential contribution of the identified risk genes to the disease.

### Tissue and developmental stage-specific expression of the identified ASD risk genes

**Fig. 2:**
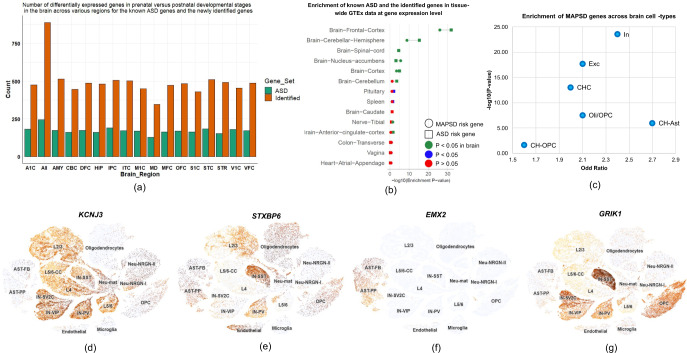
Tissue-specific enrichment of ASD risk genes and the new gene set identified by MAPSD. **a** Number of DE genes for ASD risk genes and the identified MAPSD genes across 16 brain regions in prenatal versus postnatal stages of brain development; **b** Enrichment of both new and known ASD risk genes at gene expression level in different tissues using GTEx data (Remaining insignificant tissues are not shown); **c** Enrichment statistics of the MAPSD identified genes across six cell-types in the brain in scRNA-seq data from normal human brain; **d**–**g** t-SNE plots for four genes being identified by MAPSD to be DE in distinct cell-types between normal and ASD brains. Acronyms: Amygdaloid complex, posterior (caudal) superior temporal cortex (area 22c) (STC), anterior (rostral) cingulate (medial prefrontal) cortex (MFC), dorsolateral prefrontal cortex (DFC), orbital frontal cortex (OFC), inferolateral temporal cortex (area TEv, area 20) (ITC), hippocampus, ventrolateral prefrontal cortex (VFC), primary auditory cortex (A1C), primary visual cortex (V1C), striatum (STR), primary motor-sensory cortex (M1C), posteroventral (inferior) parietal cortex (IPC), primary somatosensory cortex (S1C), cerebellum (CB), and mediodorsal nucleus of thalamus, AST-FB: fibrous astrocytes, AST-PP: protoplasmic astrocytes; OPC: oligodendrocyte precursor cells, IN-PV: parvalbumin interneurons; IN-SST: somatostatin interneurons; IN-SV2C: SV2C interneurons; IN-VIP: vasoactive intestinal polypeptide interneurons; L2/3: layer 2/3 excitatory neurons; L4: layer 4 excitatory neurons; L5/6: layer 5/6 corticofugal projection neurons; L5/6-CC: layer 5/6 cortico-cortical projection neurons; Neu-mat: maturing neurons; Neu-NRGN-I: NRGN-expressing neurons; Neu-NRGN-II: NRGN-expressing neurons.

At the gene level, we looked on the enrichment of the identified candidate genes on various tissues in the human body as well as characterizing their expression during neurodevelopment. Using the gene expression levels on 53 tissues from the Genotype-Tissue Expression project, GTEx^47^, we mapped the ASD and MAPSD risk genes onto the GTEx data and obtained the enrichment P-values across all of the available tissues. First, for each gene, we obtained the tissue in which the gene shows the highest expression level. 14 regions related to CNS were included in this analysis including tibial nerves and 13 brain regions as follows: frontal cortex, cerebral hemisphere, cortex, spinal cord, CB, nucleus accumbens, anterior cingulate cortex, caudate, hypothalamus, substantia nigra, amygdala, putamen, and hippocampus. We found both ASD and the new gene set which had the highest signal intensities in the brain to be significantly enriched in frontal cortex, cerebral hemisphere, cortex, spinal cord, and nucleus accumbens (Fig. 2b). Notably, neither gene sets were enriched on some specific brain regions such as amygdala, hippocampus, and putamen.

Given that ASD are neurodevelopmental disorders, we were interested to check if any of the two gene sets are perturbed during the development of human brains. To do this, we used the Atlas of the Developing Human Brain (BrainSpan)^48^ on 16 brain regions including: amygdaloid complex^49^, posterior (caudal) superior temporal cortex (area 22c) (STC), anterior (rostral) cingulate (medial prefrontal) cortex (MFC), dorsolateral prefrontal cortex (DFC), orbital frontal cortex (OFC), inferolateral temporal cortex (area TEv, area 20) (ITC), hippocampus^50^, ventrolateral prefrontal cortex (VFC), primary auditory cortex (A1C), primary visual cortex (V1C), striatum (STR), primary motor-sensory cortex (M1C), posteroventral (inferior) parietal cortex (IPC), primary somatosensory cortex (S1C), CB, and mediodorsal nucleus of thalamus^51^. Then, we categorized the BrainSpan data into two large sets of prenatal and postnatal developmental stages. Prenatal stage includes: 0–12 post-conception weeks (pcw), 13–24 pcw, and 25–36 pcw. Postnatal stages include: 0–2 yr, 3–8 yr, 9–16 yr, and >17 yr. Next, we averaged the expression levels of the both gene sets across different stages of pre- and postnatal stages and looked for DE genes at FDR < 0.05 (Fig. 2a, Supplementary Table 3). A constant pattern in both gene sets were observed in which almost half of the genes are DE during transition from prenatal to postnatal stages.

We were interested in further focusing on the top DE genes in each brain region, so we obtained the genes to be DE in all of the sixteen brain regions. Next, we checked all of the MAPSD brain-specific high intensity signal genes (Supplementary Table 4). We found that 229 ASD genes (~93%) are DE during developmental phases in at least one brain region. The number of brain regions that the ASD genes were enriched for ranged from 1 to 16. Within the newly identified gene set, 714 gene (80%) were DE across various number of regions ranging from 1 to 16.

We looked for demonstrating if any of the identified genes are specific markers of brain cells in postmortem adult brains. For this, we used single-cell RNA-seq (scRNA-seq) data from Lake et al.^52^ which covers >60,000 single cells from human adult frontal cortex, visual cortex, and CB. They had categorized the clustered cells into several groups including^52^: excitatory (Exc) and inhibitory neuronal (In) subtypes in the cortex, Purkinje (Purk) neurons and cerebral granule (Gran) cells as well as non-neuronal cells such as astrocytes (Ast), microgolia (Mic), oligodendrocytes (Oli) and their precursor cells (OPCs), endothelial cells (End), and pericytes. We obtained the list of the marker genes across six cell-types where their expression levels were significantly higher compared to other cells including: Exc neurons, In neurons, cerebral hemisphere cluster cells, Oli/OPCs, cerebral hemisphere Ast (CH-Ast), and cerebral hemisphere OPCs (CH-OPCs). We found that all of the MAPSD genes with the highest signal in the brain are highly enriched in all of these sets. Yet, the enrichment degress in neuronal cells such as In (Fisher’s exact test *P* value = 3 × 10^−24^) was apparently higher than non-neuronal cells such as CH-OPCs (Fisher’s exact test *P* value = 2 × 10^−2^, Fig. 2c). The details of Fig. 2c has been provided in Supplementary Table 6. This observation provides a strong evidence on how certain cell-types are involved in the disease and what genes markers are perturbed during neurodevelopment.

We looked for enrichment of MAPSD findings across diverse cell-types in the brain at the single cell resolution. We used the identified DE genes across 17 cell-types in scRNA-seq data from a study by Velmeshev et al.^19^ to intersect with the newly identified gene set. Notably, we found 20 genes to be DE across unique cell-types between ASD and normal brain tissues (Supplementary Table 5). 15 genes (~71% of the total) were found to be DE only in neuronal cells, two genes in Mic, two genes in Ast, and two genes in endothelial (End) cells. As an example, we created t-SNE plots for four MAPSD-identified genes which were DE in Velmeshev et al. scRNA-seq data^19^ including *KCNJ3*, *STXBP6*, *EMX2*, and *GRIK1* (Fig. 2d–g, darker cells denote higher expression). These genes we significantly expressed in vasoactive intestinal polypeptide interneurons, parvalbumin interneurons, protoplasmic Ast, and vasoactive intestinal polypeptide interneurons, respectively while being downregulated in ASD individuals in the same cell-types. We queried these genes to illustrate if there were any evidences on association of these genes with ASD or other brain diseases. We found eight genes to be associated with psychiatric and developmental diseases including: *ELAVL4*, *EMX2*, *GRIA4*, *UNC13B*, *GABRG2*, *GRIK1*, *GRM3*, *KCNJ3*, and *KCNH5*. Using tri whole-exome sequencing on *GRIA4*, Martin et al.^53^ had identified de novo pathogenic variants in unrelated individuals suffering from intellectual disabilities. This gene encodes *GluR4*, an AMPA receptor subunit which is found on excitatory glutamatergic synapses^53^. Such receptors are highly abundant in the CNS and bear critical impacts on glutamatergic synapses whose functions are well-documented in learning and memory^54^. Implications of *GRM3* in SCZ has been revealed through a massive metal-analysis leading to the identification of three SNPs in this gene^55^. This gene has also been reported to represent structural defects which had been observed in SCZ and attention deficit hyperactivity disorder^56^. We found multiple evidences on association of *KCNH5*^57,58^, *KCNJ3*^59^, and *GABRG2*^60,61^ with epilepsy. Other genes are also implicated in human cortical development^62^ and maintenance of axonal and synaptic structures^63,64^. Our findings sheds light of a limited set of novel cell-specific genes with potential implications in ASD that can be further investigated to create a larger picture of ASD machinery.

### The identified risk genes demonstrate ASD-relevant phenotypes in mouse models

Generating loss-of-function mutations in the mouse genome followed by evaluation of the mutant line for developmental/neurological phenotypes is a compelling approach to test the hypothesis regarding the involvement of candidate genes in ASD^65^. We sought to evaluate how our identified list of candidate genes may predispose to phenotypic traits that are relevant to ASD. We limited our list to the 21 candidate genes which are shared in the study by Velmeshev et al.^19^ We used Mouse Genome Informatics (MGI) database^66^ and queried these candidate genes for behavioral and neurological phenotypes that are relevant to ASD. 14 out of 21 genes showed phenotype annotations related to behavioral and neurological impairments as well as phenotypes related to nervous system. 10 genes were related to both categories including: *ELAVL4*, *EMX2*, *MAGI2*, *GRIA4*, *UNC13B*, *CPE*, *GABRG2*, *GRIK1*, *GRM3*, and *MAGI1*. Five genes were related either to behavioral/neurological impairments or phenotypes related to the nervous system including: *ADGRL2*, *GPATCH8*, *KCNJ3*, *ASIC2*, and *KCNH5*. For each of these candidates, we listed behavioral and neurological phenotypes in Table 1. We found multiple autistic behavioral phenotypes to be shared by the majority of the identified risk genes. For example, abnormal startle reflex and behavior as well as abnormal anxiety-related response were shared by *EMX2*, *MAGI2*, *GABRG2*, *GRM3*, *KCNJ3*, and *KCNH5*. We observed hyperactivity/hypoactivity and impaired motor capabilities as relatively consistent behavioral patterns among these knockout mice while abnormal neuron physiology was shared predominantly as a common phenotype in the nervous system. We sought to investigate if any of these genes are associated with any Mendelian diseases. Using Online Mendelian Inheritance in Man (OMIM) database^67^, we queried the 15 genes showing phenotypic manifestations of neurological impairment in mice. We found five genes to be associated with some known Mendelian diseases including neurodevelopmental impairments seizure, and certain types of epilepsy and Parkinson’s disease (Table 1). Epilepsy is a neurological disorder characterized by seizures, cognitive impairments, and psychological abnormalities^68^. Nephrotic syndrome is a set of symptoms indicating impaired functionality of kidneys including accumulation of protein in urine, swelling in some organs, and high levels of cholesterol in blood^69,70^. In fact, these observation provides further evidences about confidence of the identified candidate genes. Similarly, we found that the majority of these putative risk genes directly impact nervous system development leading to severe changes of morphology of distinct brain regions. For instance, from knockout mice studies, we observed that dysregulation of *EMX2* leads to abnormal axon extension and cerebral cortex morphology, as well as decreased cochlear nerve composition and perturbed pallium development. In conclusion, these results signifies strong evidences with regard to association of the identified risk genes with ASD and lays a solid groundwork for trans-omic studies of neurological impairments.

**Table 1:** Neurological phenotypes of the identified risk genes in mouse models.

Candidate gene	*Phenotype*			Association with Mendelian diseases from OMIM
	Behavioral/Neurological	Nervous system	*P* value	
*ELAVL4*	Limb grasping, Impaired coordination	Abnormal neuron differentiation, Abnormal nervous system development	8.15 × 10^−14^	Parkinson’s disease
*EMX2*	Absent pinna reflex	Abnormal axon extension, Abnormal cerebral cortex morphology, Decreased cochlear nerve composition, Abnormal pallium development	NA	
*MAGI2*	Abnormal social investigation, Anhedonia, Increased anxiety-related response	Abnormal brain interneuron morphology, Reduced long-term potentiation, Enlarged lateral ventricles	NA	Nephrotic syndrome
*GRIA4*	Abnormal object recognition memory, Decreased exploration in new environment, Abnormal spatial working memory, Decreased startle reflex	Abnormal neuron physiology, Abnormal inhibitory postsynaptic potential, Abnormal glutamate-mediated receptor currents	NA	Neurodevelopmental disorder with or without seizures and gait abnormalities
*UNC13B*	Sporadic seizures	Abnormal synapse morphology	15 × 10^−20^	
*CPE*	Abnormal motor capabilities/coordination/movement, Lethargy, Abnormal placing response	Abnormal hypothalamus secretion	5.85 × 10^−9^	
*GABRG2*	Impaired behavioral response to xenobiotic, Hypoactivity, Hunched posture, Increased anxiety-related response	Increased susceptibility to pharmacologically induced seizures, Abnormal brain wave pattern, Abnormal neuron physiology	NA	Epilepsy with febrile seizures plus, Epileptic encephalopathy
*GRIK1*	Increased chemical nociceptive threshold	Abnormal glutamate-mediated receptor currents, Enhanced long-term potentiation	2.95 × 10^−5^	
*GRM3*	Abnormal anxiety-related response	Abnormal astrocyte physiology, Abnormal neuron physiology	NA	
*ADGRL2*	Hypoactivity		NA	
*GPATCH8*		Abnormal neuron physiology	NA	
*KCNJ3*	Abnormal behavior, Limb grasping, Hyperactivity		2.41 × 10^−6^	
*ASIC2*		Abnormal brainstem morphology, Abnormal nervous system electrophysiology	NA	
*KCNH5*	Abnormal startle reflex, Increased thigmotaxis		NA	

### The identified risk genes are potential regulators of known ASD risk genes

**Fig. 3:**
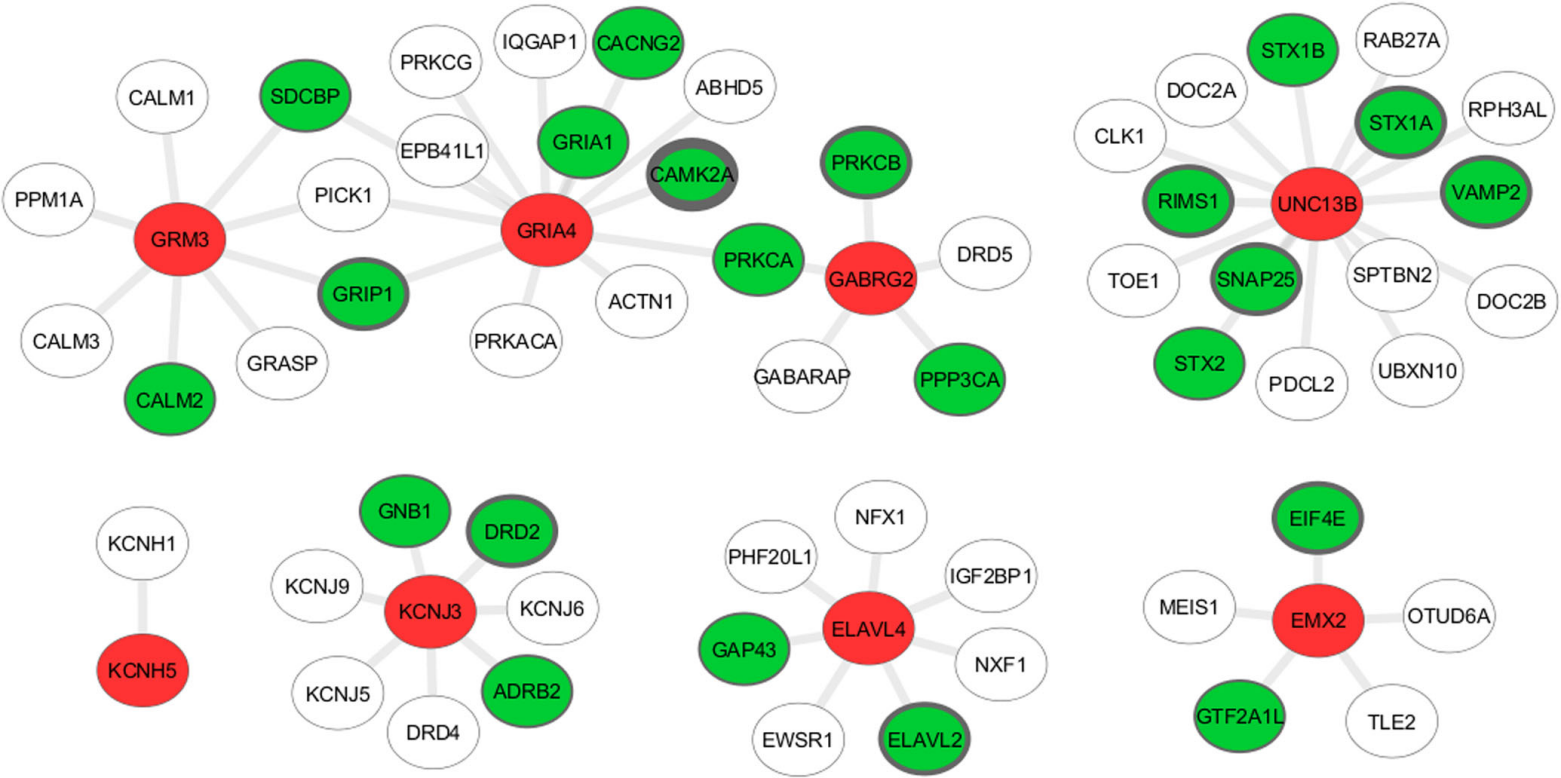
Modules of the identified ASD risk genes in the PPI network used for signal diffusion. Red nodes denote the new potential risk genes and green nodes represent existing ASD-associated risk genes. Width of borders of the green nodes represent the number of evidences available on this gene regarding its association with ASD in that thicker boundaries indicate larger evidence.

There have been thousands of genes identified to be associated with the ASD risk. Although remarkable, they do not provide insights if they converge in certain pathways of submodules of a large regulatory network. To shed light on possible concentration of ASD risk genes within PPI networks, we looked for the topological structure of submodules of the global PPI network around the identified risk genes. In the previous section, we illustrated eight genes which have not been implicated in ASD and were not available in SFARI gene list^71^. We extracted these genes and all of their neighbors within the network (Fig. 3). They bear 61 interactions in total covering 57 genes. Notably, 22 genes (%39) of the entire neighboring genes are existing ASD risk genes. We found *GRIA4* to share 13 interaction among which 6 genes are known ASD loci including *GRIA1*, *CACNG2*, *CAMK2A*, *PRKCA*, *GRIP1*, and *SDCBP* while 60% of *GABRG2* interacting genes were found to be known disease risk loci. Similarly, *UNC13B* was directly connected to 15 genes including 6 known ASD risk genes (Fig. 3). We hypothesized that, although not directly, perturbation of our identified risk genes may potentially explain disruption of the disease associated modules and can assist in revealing a larger picture of the disease architecture.

We found three genes *GRM3*, *GRIA4*, and *GABRG2* to form an interconnected module mediated by existing risk genes. These three genes were interconnected through four genes three of which known ASD loci including *PRKCA*, *SDCBP*, and *GRIP1*. Given that these genes have been implicated in other neurological disorders, they may act as potential drivers of ASD, too. We looked for the initial signal levels of the ASD-associated genes connected to the abovementioned eight genes represented in green nodes in Fig. 3. Of these 22 genes, 13 genes had an initial signal intensity of 1, four genes having intensity of 2 and 3, respectively followed by a single gene with the signal level of 4. *CAMK2A* was the only high signal gene which was connected to *GRIA1*. All of the genes connecting *GRM3*, *GRIA4*, and *GABRG2* had an initial signal of 1. Large fraction of low-signal ASD genes is a testament to importance of aggregating small-effect size disease loci in order to amplify low signal intensities to unmask un-explored disease drivers.

## Discussion

ASD are complex neurodevelopmental diseases with hundreds of genetic or environmental factors contributing to the evolution of the disease phenotypes. Significant endeavor in various domains such as GWAS or transcriptome studies have enriched our understanding of molecular mechanisms that may cause or contribute to the development of the disease. However, the majority of the research have focused on nucleic acids (genome and transcriptome studies) where proteomics has garnered less attention. At the proteome domain, there are several important aspects in ASD domain which have not been addressed such as biochemical and biophysical properties of proteins as well as their tissue/cell-specific characteristics which may affect PPIs and that how they can contribute to the disease onset and progression. In this study, using a novel single cell-based systems biology approach, we modeled intra-cellular protein trafficking patterns as well as subcellular localization of the proteins in tens of different tissues. We identified a new set of disease susceptibility genes followed by extensive examination of these genes to gather additional support of the findings.

With the advent of single cell-based technologies, exciting opportunities have been created for more accurate investigation of the disease machinery. Launching large-scale proteomic projects such as the Human Protein Atlas paved the way to make novel discoveries by modeling mutual correspondence between genome, transcriptome, and proteome. In this paper, we collected all of the available genomic, transcriptomic, and proteomic data on ASD followed by leveraging the single cell protein abundances from the Human Protein Atlas project. These multi-omic data were then fed to the MAPSD, a novel network-based systems biology method which models biophysical and biochemical properties of proteins including tissue/cell-specific protein abundances as well as subcellular localization of proteins in distinct cellular micro-domains. Our findings led to the identification of a list of new susceptibility genes that may contribute to the disease pathogenesis. Based on our analysis, the algorithm converged at the diffusion time *t* = 4. The final signal intensities generated by MAPSD are real numbers and are calculated for every gene (*n* = 16,185) in the network across all the tissues and cell-types. Then, the genes with the highest signal intensities in brain-related tissues or cell-types are picked as susceptible risk genes.

A key observation being made was the enrichment of known ASD genes in neuronal cells within the human cerebral cortex. This observation is in line with the previous findings regarding associations between cortices such as prefrontal cortex and anterior cingulate cortex with ASD^72^. These regions have been recognized to play a key role in critical cognitive processes such as decision making and motivation^73,74^. An important finding was the similarity of enrichment patterns of known ASD risk genes and the newly identified genes by MAPSD. In addition, we found that the encoded proteins of the new candidate genes highly co-localize in certain cellular micro-domains including: cytosol, nucleoplasm, nucleus, and vesicles. These micro-domains have been indicated to be impacted during neurodevelopment leading to autistic phenotypes^41,75^. Applying MAPSD to the collected data, we observed that the identified candidate genes resemble the same localization patterns as known ASD risk genes. This creates a certain level of confidence about potential association of these genes with ASD which made us put them under extra tests. Given that ASD are a clinically heterogeneous class of neurodevelopmental disorders^76^, we were interested in studying the expression variations of these genes over the course of neurodevelopment. Considering 16 brain regions from BrainSpan, we showed that over half of the known ASD genes as well as the new identified candidate loci are DE between prenatal and postnatal stages. We found that the disrupted biological pathways by the candidate risk genes show an overlap with previous findings such as Glutamatergic Synapse, Cholinergic Synapse, Embryonic and Cardiac Development as well as signaling pathways such as Insulin and Neurotrophin Signaling pathways^77^. To further investigate the list of the identified candidate risk genes, we obtained the list of ASD-associated genes by Ramaswamy et al.^26^ which contained an expanded list of 5439 dysregulated genes in ASD. Comparing our findings with this set of genes, we observed a significant overlap (Fisher’s exact test *P* value = 8 × 10^−8^, *n* = 171). We sought to check if the overlapping gene set is enriched for any biological functions. We conducted a pathway enrichment analysis on the list of overlapping genes and observed multiple neuronal-related pathways including: retrograde endocannabinoid signaling (*P* = 5.5 × 10^−3^, ratio = 1.7), circadian entertainment (*P* = 8 × 10^−3^, ratio = 1.6), neuroactive ligand-receptor interaction (*P* = 0.01, ratio = 1.5), and glutamatergic synapse (*P* = 0.01, ratio = 1.5). Such an observation serves as additional evidence regarding biological relevance of the outcomes of this research.

Using the gene expression data on 53 human tissues from the GTEx consortium, we made critical findings regarding spatial enrichment of ASD risk genes and the new identified loci. We showed that known ASD risk genes are highly expressed in specific brain regions including frontal cortex, cerebellar hemisphere, and nucleus accumbens. For the purpose of comparison, we repeated the same analysis of the new set of genes and observed an almost similar enrichment levels in the same areas. The only brain region where the new candidate gene set did not pass the significance threshold is CB where ASD risk genes showed a borderline statistical significance. Diving deep into the scRNA-seq from human brain tissue also further showed that a large fraction of the identified risk genes are in fact signature markers of inhibitory and excitatory neurons. An important discovery of this study was further validated by the scRNA-seq data from ASD brain by Velmeshev et al.^19^. We showed that 21 candidate genes which have not been captured previously by bulk transcriptional experiments were actually DE in specific cellular subpopulations in the brain. We sought to investigate the effect of subcellular localization on the outcome of MAPSD. For this, we assigned a weight of 1 to all of the edges in the PPI network and ran the algorithm. 291 genes showed the highest signal intensity in the brain after signal diffusion among them 101 genes were shared with the original list of genes identified by MAPSD (*n* = 962). Then we looked at the list of the candidate genes that were found to be dysregulated in the Velmeshev’s study^19^. However no overlapping genes were observed between the MAPSD results and their list of genes while 21 genes were found to be shared by the Velmeshev’s study when incorporating the subcellular localization information indicating the importance of considering localization information when running the model. We note that the outcomes of this study are merely a result of computational modeling without a functional assessment of the risk genes in diseases etiology. Moreover, MAPSD solely relies on molecular data to characterize potential disease-associated genes while not employing paired clinical information. Therefore, this is a limitation that the users of this method should consider.

In conclusion, using a novel systems biology technique, called MAPSD, we could successfully model proteome-specific information at the single cell resolution and pinpoint intra-cellular micro-domains where ASD risk genes-encoded proteins are enriched. As the outcome of the model, we identified a list of novel candidate loci whose disruption in mouse models increasingly leads to autistic phenotypic traits. We believe that the identified ASD risk loci as well as the systems biology approach used in this study paves the way to gain further insights into the genetic architecture of the disorder.

## Methods

### Markov affinity-based proteogenomic signal diffusion (MAPSD)

MAPSD is a network-based systems biology method aimed at identifying tissue and cell-specific candidate risk genes in complex diseases. MAPSD models biochemical and biophysical properties of proteins along with other available molecular data collected by the user to create a signal intensity vector of the known signatures of the disease. Next, the created signal vector is diffused through the regulatory network or PPI network fed to the model followed by adjusting the signals given the tissue and cell-type of interest to identify candidate risk genes. MAPSD is available at https://github.com/adoostparast/MAPSD. The tissue-wide protein abundance levels and subcellular localization information are available in the MAPSD directory. MAPSD requires a list of genetic signatures (a sample is provided in the MAPSD GitHub directory) and a PPI network as well as proteomics information for adjusting the PPI networks. Users can readily use the proteomics data available at MAPSD GitHub account without any changes.

### Creating the signal vector

The initial signal matrix *S*, is an overlaid column vector which contains the cumulative levels of biological evidences such as transcriptional signatures, methylation, GWAS, etc. For each level of information for a specific gene, we add a point 1 if there was a significant hit such as an FDR threshold of 0.05 on transcriptome signals and 5 × 10^−8^ for GWAS loci. To create *S*, first we introduce evidence matrix *E*_*G×L*_ where *G* denotes the total number of genes and *L* is the number of omics data layers. Therefore:

$$\left\{ {\begin{array}{*{20}{c}} {E_{ij} = 1;if\;for\;gene\;i\;there\;is\;an\;evidence\;in\;layer\;j} \\ {E_{ij} = 0;Otherwise} \end{array}} \right.$$

Next, using *E*, we can create *S* as follows: $S_i = \mathop {\sum}\nolimits_{j = 1}^L {e_{ij}}$. As example if a gene *i* is DE and differentially methylated, then *S*_*i*_ = 2. To avoid generating spurious signals, we should make sure that the data being collected to create the signal vector have been generated from the same tissue or appropriate surrogate tissues.

### Data sets used in the study

SFARI Gene set 3.0 is used for collecting genetic signatures of ASD which are categorized as rare single gene mutation, functional mutations, common variants, and syndromic loci. SFARI gene list^71^ included 1089 genes. Pre-calculated list of DE and differentially spliced ASD genes were obtained from PsychENCODE project^36^. This data are available on PsychENCODE Knowledge Portal under Synapse ID syn4587609.

To study the expression patterns of genes during neurodevelopment, we download the RNA-seq data from the Atlas of the Developing Human Brain (BrainSpan)^48^ and calculated the DE genes between prenatal and postnatal stages using *t*-test followed by false discovery rate correction (FDR < 0.05). To characterize spatial expression of genes in different brain regions, we used GTEx V8^78^ and downloaded the processed RNA-seq data on the human brain. To characterize the ASD manifestations of disrupting the candidate risk genes, we used MGI database^66^. MGI contains knockout mouse models to investigate the phenotypic similarities between human patients and mouse models. We queried the candidate risk gene in MGI search tab and downloaded the resulting output.

Protein-protein interactions were collected from three sources including: PICKLE 2.3^33,34^, The Human Reference Interactome^27^, and human Interactome Database^35^. Next, duplicate interactions were removed leading to 232,801 unique interactions to be used in the study.

## Supplementary information

Supplementary Figure 1

Supplementary Table 1

Supplementary Table 2

Supplementary Table 3

Supplementary Table 4

Supplementary Table 5

Supplementary Table 6

## Data Availability

All the generated results in this study are available in supplementary tables.

## Code availability

The code for running MAPSD is available at https://github.com/adoostparast/MAPSD.

## References

[1] American Psychiatric Association & American Psychiatric Association. DSM-5 Task Force. *Diagnostic and statistical manual of mental disorders: DSM-5*, xliv, 947 p. (American Psychiatric Association, Washington, D.C., 2013).

[2] Maenner MJ (2020). Prevalence of Autism Spectrum Disorder Among Children Aged 8 Years - Autism and Developmental Disabilities Monitoring Network, 11 Sites, United States, 2016. MMWR Surveill. Summ..

[3] Baio J (2018). Prevalence of Autism Spectrum Disorder Among Children Aged 8 Years — Autism and Developmental Disabilities Monitoring Network, 11 Sites, United States, 2014. MMWR Surveill. Summaries.

[4] de la Torre-Ubieta L, Won H, Stein JL, Geschwind DH (2016). Advancing the understanding of autism disease mechanisms through genetics. Nat. Med..

[5] Doostparast Torshizi A, Duan J, Wang K (2018). Transcriptional network analysis on brains reveals a potential regulatory role of PPP1R3F in autism spectrum disorders. BMC Res Notes..

[6] Satterstrom FK (2020). Large-Scale Exome Sequencing Study Implicates Both Developmental and Functional Changes in the Neurobiology of Autism. Cell.

[7] Zhang Y (2020). Genetic evidence of gender difference in autism spectrum disorder supports the female-protective effect. Transl. Psychiatry.

[8] Werling DM, Parikshak NN, Geschwind DH (2016). Gene expression in human brain implicates sexually dimorphic pathways in autism spectrum disorders. Nat. Commun..

[9] Geschwind DH (2008). Autism: many genes, common pathways?. Cell.

[10] Parikshak NN (2013). Integrative functional genomic analyses implicate specific molecular pathways and circuits in autism. Cell.

[11] Levinson DF (2012). Genome-wide association study of multiplex schizophrenia pedigrees. Am. J. Psychiatry.

[12] Gaugler T (2014). Most genetic risk for autism resides with common variation. Nat. Genet..

[13] Grove J (2019). Identification of common genetic risk variants for autism spectrum disorder. Nat. Genet..

[14] Iossifov I (2014). The contribution of de novo coding mutations to autism spectrum disorder. Nature.

[15] Borgmann-Winter KE (2019). The proteome and its dynamics: a missing piece for integrative multi-omics in schizophrenia. Schizophrenia Res.

[16] De Rubeis S (2014). Synaptic, transcriptional and chromatin genes disrupted in autism. Nature.

[17] Michaelson JJ (2012). Whole-genome sequencing in autism identifies hot spots for de novo germline mutation. Cell.

[18] Hu VW (2009). Gene expression profiling differentiates autism case-controls and phenotypic variants of autism spectrum disorders: evidence for circadian rhythm dysfunction in severe autism. Autism Res..

[19] Velmeshev D (2019). Single-cell genomics identifies cell type-specific molecular changes in autism. Science.

[20] Quesnel-Vallieres M, Weatheritt RJ, Cordes SP, Blencowe BJ (2019). Autism spectrum disorder: insights into convergent mechanisms from transcriptomics. Nat. Rev. Genet..

[21] Broek JA, Guest PC, Rahmoune H, Bahn S (2014). Proteomic analysis of post mortem brain tissue from autism patients: evidence for opposite changes in prefrontal cortex and cerebellum in synaptic connectivity-related proteins. Mol. Autism.

[22] Ngounou Wetie AG (2015). A Pilot Proteomic Analysis of Salivary Biomarkers in Autism Spectrum Disorder. Autism Res..

[23] Nagaraj N (2011). Deep proteome and transcriptome mapping of a human cancer cell line. Mol. Syst. Biol..

[24] Schwanhausser B (2011). Global quantification of mammalian gene expression control. Nature.

[25] Yugi K, Kubota H, Hatano A, Kuroda S (2016). Trans-Omics: How To Reconstruct Biochemical Networks Across Multiple ‘Omic’ Layers. Trends Biotechnol..

[26] Ramaswami G (2020). Integrative genomics identifies a convergent molecular subtype that links epigenomic with transcriptomic differences in autism. Nat. Commun..

[27] Luck K (2020). A reference map of the human binary protein interactome. Nature.

[28] Ben-David E, Shifman S (2013). Combined analysis of exome sequencing points toward a major role for transcription regulation during brain development in autism. Mol. Psychiatry.

[29] Voineagu I (2011). Transcriptomic analysis of autistic brain reveals convergent molecular pathology. Nature.

[30] Sakai Y (2011). Protein interactome reveals converging molecular pathways among autism disorders. Sci. Transl. Med..

[31] Doostparast Torshizi A, Duan J, Wang K (2020). Cell-Type-Specific Proteogenomic Signal Diffusion for Integrating Multi-Omics Data Predicts Novel Schizophrenia Risk Genes. Patterns (N Y).

[32] Doostparast Torshizi A (2019). Deconvolution of transcriptional networks identifies TCF4 as a master regulator in schizophrenia. Sci. Adv..

[33] Gioutlakis A, Klapa MI, Moschonas NK (2017). PICKLE 2.0: A human protein-protein interaction meta-database employing data integration via genetic information ontology. PLoS ONE.

[34] Klapa MI, Tsafou K, Theodoridis E, Tsakalidis A, Moschonas NK (2013). Reconstruction of the experimentally supported human protein interactome: what can we learn?. BMC Syst. Biol..

[35] Rolland T (2014). A proteome-scale map of the human interactome network. Cell.

[36] Gandal MJ (2018). Transcriptome-wide isoform-level dysregulation in ASD, schizophrenia, and bipolar disorder. Science.

[37] Uhlen M (2015). Proteomics. Tissue-based map of the human proteome. Science.

[38] Thul PJ (2017). A subcellular map of the human proteome. Science.

[39] Behesti H (2018). ASTN2 modulates synaptic strength by trafficking and degradation of surface proteins. Proc. Natl Acad. Sci. USA.

[40] Adamsen D (2014). Autism spectrum disorder associated with low serotonin in CSF and mutations in the SLC29A4 plasma membrane monoamine transporter (PMAT) gene. Mol. Autism.

[41] Wegiel J (2015). Neuronal nucleus and cytoplasm volume deficit in children with autism and volume increase in adolescents and adults. Acta Neuropathol. Commun..

[42] Kurochkin I (2019). Metabolome signature of autism in the human prefrontal cortex. Commun. Biol..

[43] Liao Y, Wang J, Jaehnig EJ, Shi Z, Zhang B (2019). WebGestalt 2019: gene set analysis toolkit with revamped UIs and APIs. Nucleic Acids Res.

[44] Wen Y, Alshikho MJ, Herbert MR (2016). Pathway Network Analyses for Autism Reveal Multisystem Involvement, Major Overlaps with Other Diseases and Convergence upon MAPK and Calcium Signaling. PLoS ONE.

[45] Horder J (2018). Glutamate and GABA in autism spectrum disorder-a translational magnetic resonance spectroscopy study in man and rodent models. Transl. Psychiatry.

[46] Rojas DC (2014). The role of glutamate and its receptors in autism and the use of glutamate receptor antagonists in treatment. J. Neural Transm. (Vienna).

[47] Ardlie KG (2015). The Genotype-Tissue Expression (GTEx) pilot analysis: Multitissue gene regulation in humans. Science.

[48] Miller JA (2014). Transcriptional landscape of the prenatal human brain. Nature.

[49] Ramasamy A (2014). Genetic variability in the regulation of gene expression in ten regions of the human brain. Nat. Neurosci..

[50] Ritchie ME (2015). limma powers differential expression analyses for RNA-sequencing and microarray studies. Nucleic Acids Res..

[51] Lessel D (2018). BCL11B mutations in patients affected by a neurodevelopmental disorder with reduced type 2 innate lymphoid cells. Brain.

[52] Lake BB (2018). Integrative single-cell analysis of transcriptional and epigenetic states in the human adult brain. Nat. Biotechnol..

[53] Martin S (2017). De Novo Variants in GRIA4 Lead to Intellectual Disability with or without Seizures and Gait Abnormalities. Am. J. Hum. Genet..

[54] Riedel G, Platt B, Micheau J (2003). Glutamate receptor function in learning and memory. Behav. Brain Res..

[55] Saini SM (2017). Meta-analysis supports GWAS-implicated link between GRM3 and schizophrenia risk. Transl. Psychiatry.

[56] Hadley D (2014). The impact of the metabotropic glutamate receptor and other gene family interaction networks on autism. Nat. Commun..

[57] Niday Z, Tzingounis AV (2018). Potassium Channel Gain of Function in Epilepsy: An Unresolved Paradox. Neuroscientist.

[58] Veeramah KR (2013). Exome sequencing reveals new causal mutations in children with epileptic encephalopathies. Epilepsia.

[59] Choi BJ (2014). Genetic association of KCNA5 and KCNJ3 polymorphisms in Korean children with epilepsy. Mol. Cell. Toxicol..

[60] Kang JQ, Macdonald RL (2016). Molecular Pathogenic Basis for GABRG2 Mutations Associated With a Spectrum of Epilepsy Syndromes, From Generalized Absence Epilepsy to Dravet Syndrome. JAMA Neurol..

[61] Shen D (2017). De novo GABRG2 mutations associated with epileptic encephalopathies. Brain.

[62] Bayatti N (2008). Progressive loss of PAX6, TBR2, NEUROD and TBR1 mRNA gradients correlates with translocation of EMX2 to the cortical plate during human cortical development. Eur. J. Neurosci..

[63] Ogawa Y (2018). Elavl3 is essential for the maintenance of Purkinje neuron axons. Sci. Rep..

[64] Kraushar ML (2014). Temporally defined neocortical translation and polysome assembly are determined by the RNA-binding protein Hu antigen R. Proc. Natl Acad. Sci. USA.

[65] Silverman JL, Yang M, Lord C, Crawley JN (2010). Behavioural phenotyping assays for mouse models of autism. Nat. Rev. Neurosci..

[66] Bult CJ (2019). Mouse Genome Database (MGD) 2019. Nucleic Acids Res..

[67] Amberger JS, Bocchini CA, Scott AF, Hamosh A (2019). OMIM.org: leveraging knowledge across phenotype-gene relationships. Nucleic Acids Res..

[68] Devinsky O (2018). Epilepsy. Nat. Rev. Dis. Prim..

[69] Kodner C (2016). Diagnosis and Management of Nephrotic Syndrome in Adults. Am. Fam. Phys..

[70] Mahalingasivam V, Booth J, Sheaff M, Yaqoob M (2018). Nephrotic syndrome in adults. Acute Med..

[71] Abrahams BS (2013). SFARI Gene 2.0: a community-driven knowledgebase for the autism spectrum disorders (ASDs). Mol. Autism.

[72] Guo B (2019). Anterior cingulate cortex dysfunction underlies social deficits in Shank3 mutant mice. Nat. Neurosci..

[73] Holroyd CB, Yeung N (2012). Motivation of extended behaviors by anterior cingulate cortex. Trends Cogn. Sci..

[74] Apps, Matthew AJ, Rushworth, Matthew FS, Chang, Steve WC (2016). The Anterior Cingulate Gyrus and Social Cognition: Tracking the Motivation of Others. Neuron.

[75] Giulivi C (2010). Mitochondrial dysfunction in autism. JAMA.

[76] Iakoucheva LM, Muotri AR, Sebat J (2019). Getting to the Cores of Autism. Cell.

[77] Pinto D (2014). Convergence of genes and cellular pathways dysregulated in autism spectrum disorders. Am. J. Hum. Genet.

[78] Consortium, G. T. (2020). The GTEx Consortium atlas of genetic regulatory effects across human tissues. Science.

