# Supplementary material for: Tissue-wide cell-specific proteogenomic modeling reveals novel candidate risk genes in autism spectrum disorders
Source: NPJ Syst Biol Appl. 2022 Sep 6;8:31. doi: 10.1038/s41540-022-00243-8 (PMC9448731; doi:10.1038/s41540-022-00243-8)
Supplement: Supplementary file 1 — Supplementary Figure 1 [file 41540_2022_243_MOESM1_ESM.pdf]

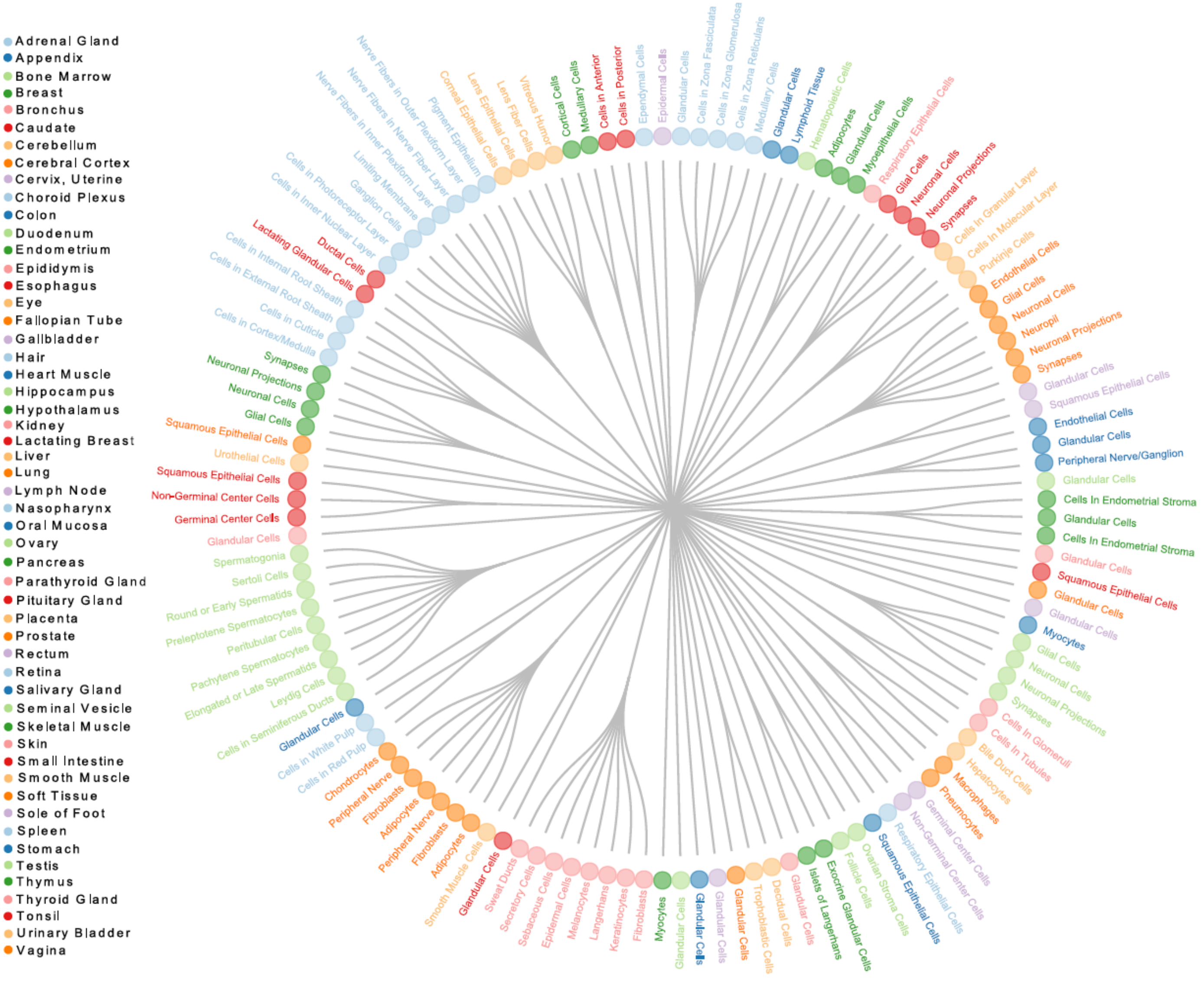

(a)

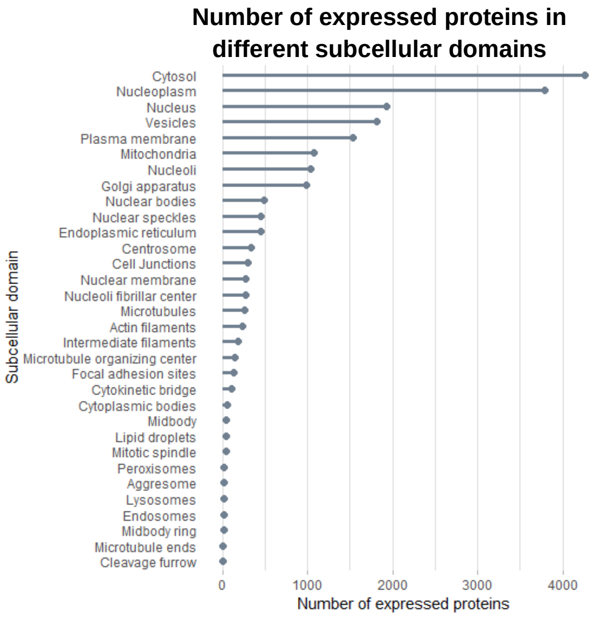

(b)

Supplementary Figure 1. The list of cell-types and tissues used in this study. (a) 131 combination of cell-types and tissues. Each color denotes a tissue and the forks for each color represents their corresponding cell-types in this study; (b) The list of subcellular domains in this study followed by the number of proteins being expressed in each subcellular domain.
